# “Green” Synthesis of Cytotoxic Silver Nanoparticles Based on Secondary Metabolites of Lavandula Angustifolia Mill.

**DOI:** 10.32607/20758251-2019-11-2-47-53

**Published:** 2019

**Authors:** M. M. Belova, V. O. Shipunova, P. A. Kotelnikova, A. V. Babenyshev, E. A. Rogozhin, M. Yu. Cherednichenko, S. M. Deyev

**Affiliations:** Russian State Agrarian University-Moscow Timiryazev Agricultural Academy, Timiryazevskaya Str. 49 , Moscow, 127550, Russia; Shemyakin-Ovchinnikov Institute of Bioorganic Chemistry of the Russian Academy of Sciences, GSP-7, Miklukho-Maklaya Str. 16/10, Moscow, 117997, Russia; Moscow Institute of Physics & Technology, Kerchenskaya Str. 1 “A”, Moscow, 117303, Russia; National Research Nuclear University MEPhI (Moscow Engineering Physics Institute), Kashirskoe sh. 31, Moscow, 115409, Russia; Sechenov First Moscow State Medical University, Trubetskaya Str., 8-2, Moscow, 119991, Russia

**Keywords:** green synthesis, silver nanoparticles, secondary metabolites, lavender

## Abstract

In this study, we used “green” synthesis to prepare silver nanoparticles (NPs) from aqueous plant and callus extracts of the narrow-leaved lavender *Lavandula angustifolia *Mill. 35.4 ± 1.6 nm and 56.4 ± 2.4 nm nanoparticles, colloidally stable in phosphate-buffered saline, were synthesized using the plant extract and the callus extract, respectively. NPs were characterized by spectrophotometry, dynamic light scattering, and scanning electron microscopy. We studied the dynamics of the nanoparticle synthesis and evaluated the cytotoxic properties of the plant extract-based NPs. Modification of NPs with bovine serum albumin demonstrated that blockage of the nanoparticle surface completely suppressed NP cytotoxic activity *in vitro*. The synthesized NPs possess localized surface plasmon resonance properties and are of small sizes, and their surface can be modified with protein molecules, which makes them promising agents for cancer theranostics.

## INTRODUCTION

Modern bionanotechnologies help unlock broad prospects for the development of new generations of drugs that can be used to combat socially impactful diseases. Bionanotechnological means and methods enable the creation of various nanostructures that serve as effective tools for the therapy and diagnosis (theranostics) of various diseases, in particular cancers.

The development of theranostic methods is based on multifunctional agents combining diagnostic and therapeutic functions [1–5]. These agents include metallic nanoparticles (NPs) with localized surface plasmon resonance (LSPR) properties [6]. The high chemical surface activity of these nanoparticles allows one to modify them by targeting agents for delivery to target cells, while LSPR makes these nanoparticles suitable for both detection and selective hyperthermal destruction of cells [7, 8]. The “green” synthesis that implies an environmentally friendly production of particles without the use of aggressive toxic and expensive substances is an alternative, economically more profitable, and environmentally safe way to prepare nanostructures compared to traditional physicochemical methods for NP synthesis, which are often expensive, labor-intensive, and not environmentally friendly [9].

In the “green” synthesis, secondary plant metabolites (SMs) are widely used as reducing agents [10–12]. They are particularly promising in “green” synthesis thanks to their low cost of production, short-term synthesis, and biosafety. Also, *in vitro *cultivation of plants enables one to scale up the production of necessary substances, because these methods yield large amounts of standardized plant materials within a short time period and produce desired SMs all year round.

The development of a successful nano-agent for effective action on cancer cells relies on a number of parameters, such as size, composition, coating, other physicochemical properties, blood-circulation characteristics, etc. Biocompatibility is one of the most essential parameters affecting the fundamental possibility of using the drug *in vivo*. NPs produced by “green” synthesis often have higher biocompatibility thanks to the use of natural substances with the necessary biological activity (noble metals, SMs, proteins), which is successfully used for various *in vitro *and *in vivo *studies. These particles are considered as promising for theranostics [13, 14].

In this work, we used “green” synthesis to prepare silver NPs based on aqueous extracts of the narrow-leaved lavender (*Lavandula angustifolia *Mill). The dynamics of nanoparticle synthesis and NP cytotoxic properties before and after surface modification were studied *in vitro*.

## EXPERIMENTAL

**Introduction of the plant material into culture *in vitro***

Narrow-leaved lavender (*L. angustifolia *Mill., Munstead, Lamiaceae Mart.) seeds were sterilized with a 5% sodium hypochlorite solution for 10 min. After sterilization, the seeds were washed twice in sterile distilled water and placed in Petri dishes with a Murashige and Skoog (MS) hormone-free medium [15]. Control seeds were germinated in non-sterile conditions on filter paper moistened with distilled water. Seed germination capacity was evaluated on the 15^th^ day according to GOST 30556-98 [16]. Three weeks after planting, the seedlings were replanted into containers with the MS medium for further development.

**Clonal micropropagation**

Plants with a height of 10 cm (4–6 nodes) were cut into cuttings (a node with internode parts) and propagated in two stages: planting in the MS medium supplemented with 0.5 mg/L thidiazuron (TDZ) to stimulate aerial part growth and then replanted in ¼ MS medium with addition of 0.2 mg/L α-naphthylacetic acid to induce rhizogenesis [17].

**Callusogenesis induction**

Stem explants were placed in the MS medium supplemented with 0.5 mg/L of 2,4-dichlorophenoxyacetic acid (2,4-D). Callusogenesis was induced using previously *in vitro *cultivated plants.

**Preparation of aqueous extracts**

Aqueous extracts were prepared from the aerial part of the aseptic plants and the callus. The plant material, frozen in liquid nitrogen, was homogenized in a mortar. After achieving room temperature, the homogenate was added with distilled water at a 1 : 3 ratio. The mixture was placed in a water bath and boiled for 30 min [18]; the extract was filtered and centrifuged at 20,000 g for 60 min; the supernatant was collected and used for the synthesis of nanoparticles.

**Isolation of predominant fractions of the plant extract**

The aqueous lavender extract was investigated using analytical chromatography. Chromatograms of the aqueous lavender plant and callus extracts were analyzed at three wavelengths (214, 280, and 320 nm). The fractions corresponding to the maximum peaks (denoted by numbers in *Fig. 5A*) where dried using a lyophilizer and dissolved in a RPMI-1640 medium supplemented with 10% fetal bovine serum and used to evaluate cytotoxicity.

**Nanoparticle synthesis**

Silver nanoparticles were prepared using “green” synthesis by mixing 50 μL of a silver nitrate solution in water (1 g/L) and 50 μL of either the lavender plant or callus extracts in a concentration range of 0.5% to 30%. During particle synthesis, absorption spectra at 350–800 nm were measured at four time points (30, 60, 150, and 240 min) using an Infinite M100 Pro plate reader (Tecan, Austria). The efficiency of NP synthesis was evaluated by the LSPR peak intensity. The surface plasmon resonance peak is considered to be a qualitative criterion for the presence of metallic NPs in a system [19, 20].

The morphology of the synthesized nanoparticles was investigated by scanning electron microscopy at an accelerating voltage of 10 kV on a MAIA3 Tescan microscope (Czech Republic).

**Nanoparticle Modification**

NPs were modified with bovine serum albumin (BSA) by sorption of the protein on the particle surface. The efficiency of NP modification was indirectly confirmed by measuring their hydrodynamic size. Particle size was determined by dynamic light scattering on a Zetasizer Nano ZS analyzer (Malvern Instruments, Ltd).

**Analysis of cytotoxic properties**

The cytotoxic properties of the plant extract, its predominant fractions, and extract-based NPs were analyzed before and after stabilization with BSA using a standard MTT test. The analysis was performed on cell lines of different origins: Chinese hamster ovary (CHO) cells, human breast adenocarcinoma (SK-BR-3), human ovarian adenocarcinoma (SKOV3-1ip), as well as on a SKOV-kat line transfected with the Katushka red fluorescent protein for intravital monitoring of malignant tumor development *in vivo *in model laboratory animals [21].

## RESULTS AND DISCUSSION

Silver nanoparticles for biomedical applications were synthesized using an aqueous extract of the narrow-leaved lavender, which is an essential oil plant widely used in the food, cosmetics, and pharmaceutical industries. The use of lavender secondary metabolites (SMs) capable of reducing metal ions from their salts is a promising, environmentally safe way to create NPs with antibacterial and cytotoxic properties. A number of nanoparticles produced via reduction of metal ions possess surface plasmon resonance properties and, therefore, are capable of heating, which may be used in cancer theranostics for tissue hyperthermia.

**Narrow-leaved lavender cell and tissue culture**

**Fig. 1:**
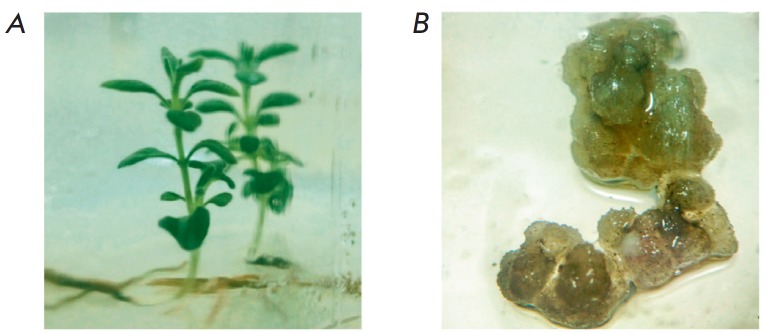
Plants (A) and callus (B) of *Lavandula angustifolia *Mill., Munstead, produced in culture *in vitro *

During *in vitro *cultivation, narrow-leaved lavender seedlings were produced (*Fig. 1A*). Seed germination upon introduction into *in vitro *culture did not differ significantly from germination in the control sample and was 80.0 ± 19.6%, which indicated the efficiency of the chosen sterilization method.

Propagation of plants on the MS medium supplemented with 0.5 mg/L TDZ resulted in seedlings with a mean height of 6.4 ± 2.1 cm. There was multiple shoot growth, which is considered to be a good indicator of efficient increase in the plant vegetative mass. Also, 4% of the cuttings had spontaneous rhizogenesis; these plants did not need further replanting.

For rooting of the remaining plants, we used a medium with low macroelement contents, ¼ MS supplemented with 0.2 mg/L α-naphthylacetic acid; in this case, the rate of rhizogenesis was 90.7–93.3%. This stage of clonal micropropagation significantly increased the efficiency of root formation in previously produced plants.

The rate of callusogenesis in stem explants was 95–99%. The callus had a loose consistency and a light green hue (*Fig. 1B*). A callus with these properties may be further used to produce a plant cell suspension, which increases the yield of SMs in *in vitro* culture.

**Nanoparticle synthesis**

**Fig. 2:**
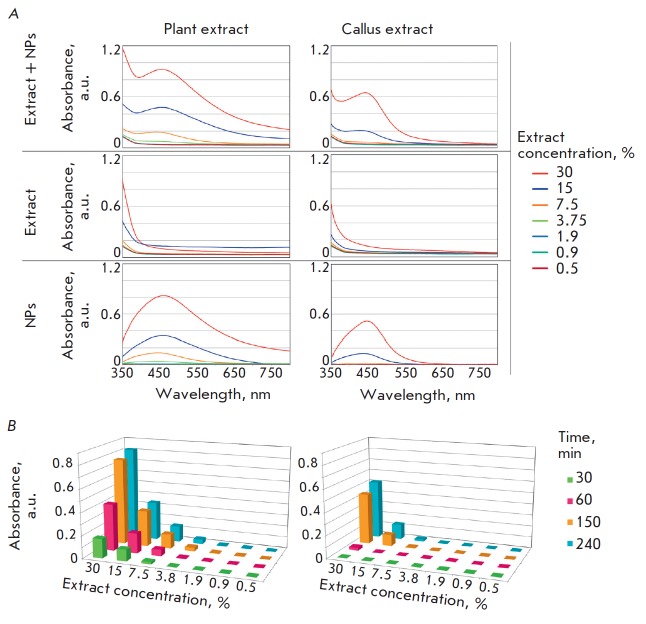
Analysis of the nanoparticle synthesis efficiency. *A *– absorption spectra of an extract and nanoparticle mixture (top panels), an extract (middle panels), and nanoparticles (bottom panels) in a range of 350–800 nm, which were produced during 240 minute incubation of an aqueous silver nitrate solution (1 g/L) and plant (left panels) or callus (right panels) extracts at 0.5–30% concentrations (shown in colors). *B *– intensity of LSPR peaks during the synthesis of silver NPs, depending on the concentrations of plant (left panel) and callus (right panel) extracts and the time of incubation (shown in colors) of extracts and an aqueous silver nitrate solution (1 g/L)

Particles were produced by long-term incubation of a silver nitrate solution and lavender plant and callus extracts as described in the Experimental section. The efficiency of the synthesis of nanoparticles exhibiting a LSPR peak was quantitatively assessed by spectrophotometry, which enables identification of the LSPR peak and measurement of its intensity. The spectra of mixtures of the silver nitrate solution and lavender plant and callus extracts obtained at different time points (*Fig. 2*) demonstrate a monotonically increasing relationship between the NP sample absorbance at the LSPR peak wavelength and the extract concentration, as well as the silver salt and extract incubation time. The highest sample absorbance was observed for the synthesis using the plant extract (30%) at 240 min incubation and amounted to 0.82, which was 1.6-fold higher than a similar indicator for the callus extract (*Fig. 2*). Further, a 7.5% extract was used for NP synthesis, because a rather intense plasmon resonance peak was observed at this concentration, confirming the formation of nanostructures; also, silver salt excess was maintained in the solution.

Further, NP colloidal stability was studied. The particles showed aggregation and sedimentation stability in phosphate-buffered saline for a long time (monitoring duration was 3 months) without any surface modification, which is considered to be a good indicator for the chosen synthesis method. It should be noted that metal particles in most cases require additional treatment with various stabilizers (sodium citrate, proteins, PEG, and other polymers) to provide colloidal stability in buffer solutions. The synthesized NPs may be used for subsequent modification by biologically active molecules, in particular by polypeptides recognizing cancer cells (antibodies, scaffolds), which require long-term storage in saline solutions.

**Fig. 3:**
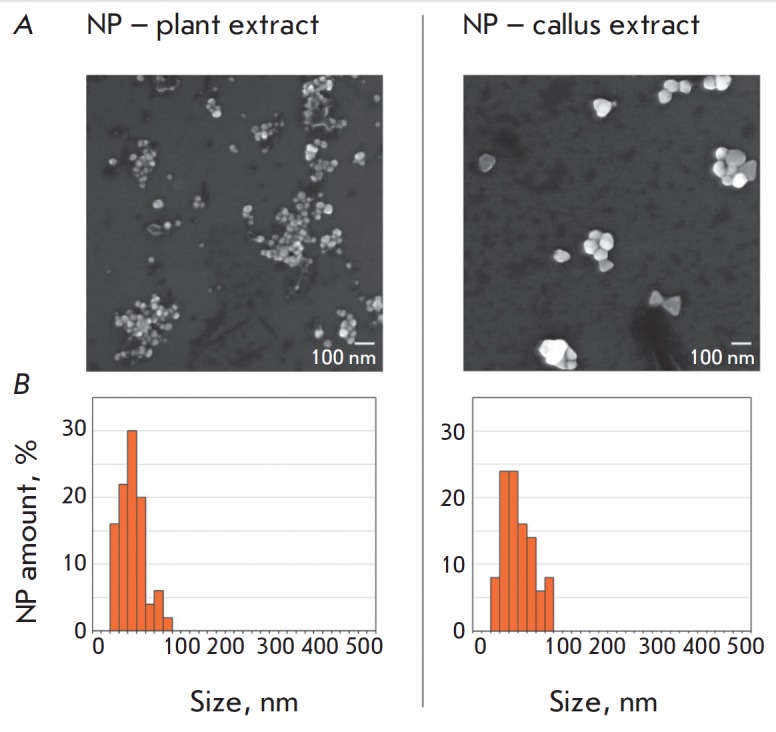
Morphological analysis of silver nanoparticles. *A *– scanning electron microscopy microphotographs of nanoparticles at an accelerating voltage of 10 kV on a MAIA3 Tescan microscope (Czech Republic). *B *– NP size distribution histograms

Processing of electron microscopy images yielded the mean size of nanoparticles: 35.4 ± 1.6 nm in synthesis with the plant extract and 56.4 ± 2.4 nm in synthesis with the callus extract (*Fig. 3B*). The NPs were mostly rounded, but some callus extract-based particles had a tetrahedron or a more complex polyhedron shape (*Fig. 3A*).

It should be noted that the size of the NPs used *in vivo *is of great importance, because it controls nanoparticle properties and affects their penetration through the blood-brain barrier [22–24].

Therefore, during the synthesis of nanoparticles, it is necessary to consider all the parameters controlling their size, as well as be capable of affecting these parameters to produce optimal size nanoparticles for successful penetration into the cell.

In further experiments, we used silver NPs produced using the plant extract, because they exceeded callus extract-based NPs in all aspects — they had a higher SPR peak intensity, a smaller size, and a more stable shape. Because application of nanoparticles for cancer theranostics purposes implies modification of their surface by various substances (antibodies, affibodies, etc.), which significantly affects the final hydrodynamic size, nanoparticles with a smaller mean diameter were chosen for the experiments.

**Analysis of NP cytotoxic properties**

To elucidate the prospects of synthesized NPs for various biomedical applications, in particular for cancer theranostics, we investigated the biocompatibility of these NPs in culture *in vitro*. A standard MTT test was used to study the effect of plant extract-based NPs and extract fractions that may affect the cytotoxicity of both the extract and the NPs.

**Fig. 4:**
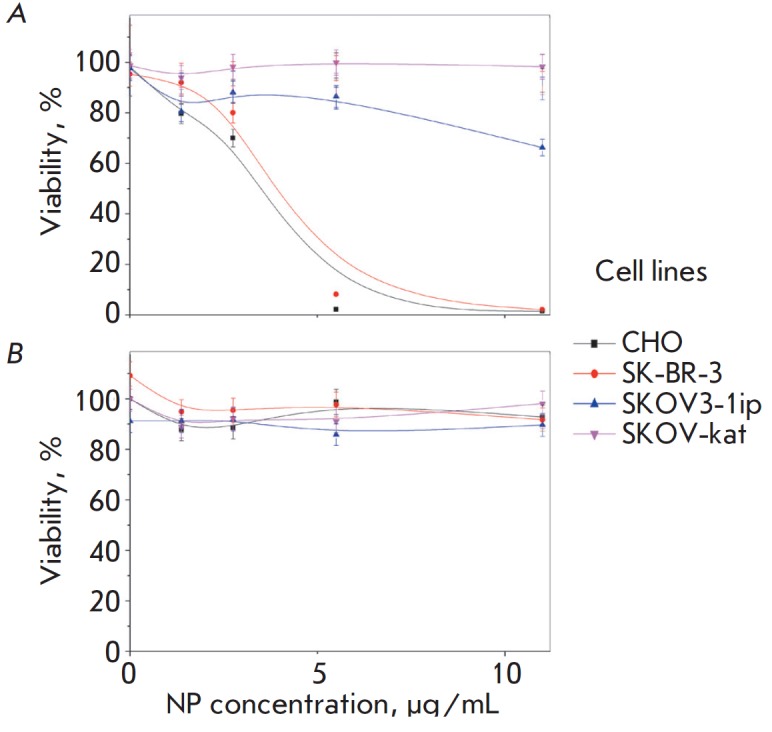
Analysis of silver nanoparticle cytotoxicity by the MTT-test. Cell viability dependence (%) for CHO, SK-BR-3, SKOV3-1ip, and SKOV-kat lines (shown in colors) on the content of silver nanoparticles in the culture medium (μg/mL) before (*A*) and after (*B*) nanoparticle stabilization with bovine serum albumin (BSA)

According to the MTT test (*Fig. 4A*), synthesized unmodified nanoparticles were more cytotoxic against CHO and SK-BR-3 cell lines than they were against SKOV3-1ip. Unmodified NPs did not affect the viability of SKOV-kat cells.

**Fig. 5:**
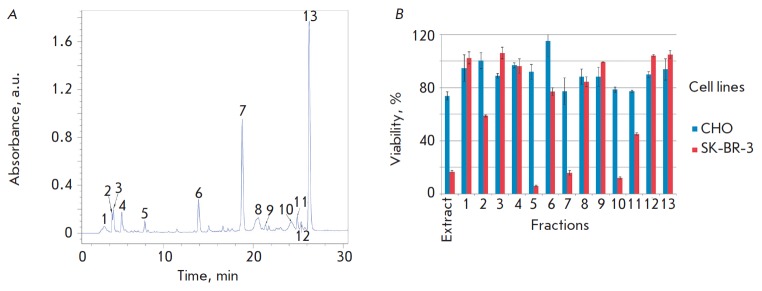
Analysis of plant extract cytotoxicity. *A *– plant extract chromatogram (λ = 280 nm). Numbers denote the absorption peaks corresponding to the predominant fractions obtained from the extract. *B *– cytotoxicity analysis of the plant extract and its predominant fractions by the MTT-test. Dependence of the viability (%) of the CHO and SK-BR-3 cell lines (shown in colors) on the contents of the plant extract and its predominant fractions (1–13) in the medium

The effect of the plant extract and its main fractions isolated by analytical chromatography on CHO and SK-BR-3 cell lines was evaluated using the MTT test. The fractions corresponding to the highest absorbance peaks at λ = 280 nm (*Fig. 5A*) were isolated. Given the data presented in *Fig. 5A*, we supposed that the cytotoxicity of the produced NPs towards these cell lines was due to the presence of biologically active substances on the NP surface; namely, secondary metabolites from the plant extract used in their synthesis. To test this hypothesis, we analyzed the cytotoxic effect of both a 1% extract and its fractions, dried and dissolved in the growth medium. According to the MTT test (*Fig. 5B*), fractions 2 and 6 exhibited significantly greater cytotoxicity towards the SK-BR-3 line but had no effect on the viability of the CHO line. Fractions 3, 9, and 12 had the opposite effect. The highest cytotoxic effect on both cell lines was exerted by the extract itself, as well as by fractions 5, 7, 10, and 11, with the effect on the viability of the SK-BR-3 cell line being more pronounced. Therefore, we may suggest that the cytotoxic properties of the plant extract and, probably, the cytotoxic properties of NPs are determined mainly by fractions 5, 7, 10, and 11.

Because NPs exerted a cytotoxic effect on some cell lines, we suggest that blockage of the particle surface with a biocompatible protein may reduce this effect. Bovine serum albumin (BSA) not affecting cell viability was chosen as a blocking protein.

The diameter of the particles after stabilization with BSA increased by 71.9 nm, on average. According to the MTT test results in culture *in vitro*, the BSA-modified nanoparticles had no cytotoxic effect on all studied cell lines (*Fig. 4B*). These findings suggest that BSA shields the surface of NPs, thereby blocking their cytotoxicity.

## CONCLUSIONS

Colloidally stable silver nanoparticles were produced by “green” synthesis using aqueous plant and callus extracts of the narrow-leaved lavender. We selected conditions for the synthesis of NPs stable in phosphate-buffered saline, which had a size of 35.4 ± 1.6 nm, optimal for application in cancer ther anostics. NPs were characterized by spectrophotometry, dynamic light scattering, and scanning electron microscopy. The cytotoxic properties of the plant extract-based particles were studied. Blockage of the NP surface with BSA was demonstrated to completely inhibit their cytotoxic effect *in vitro*. The produced NPs have a set of properties that predetermine the prospects of their use for the development of multifunctional agents combining diagnostic and therapeutic functions.

## Abbreviations

BSA: bovine serum albumin
SM: secondary metabolite
LSPR: localized surface plasmon resonance
NP: nanoparticle
MS: Murashige and Skoog medium
PEG: polyethylene glycol
TDZ: thidiazuron
